# Evidence for strong seasonality in the carbon storage and carbon use efficiency of an Amazonian forest

**DOI:** 10.1111/gcb.12375

**Published:** 2014-01-20

**Authors:** Lucy Rowland, Timothy Charles Hill, Clement Stahl, Lukas Siebicke, Benoit Burban, Joana Zaragoza-Castells, Stephane Ponton, Damien Bonal, Patrick Meir, Mathew Williams

**Affiliations:** * School of Geosciences, University of EdinburghEdinburgh, EH9 3JN, UK; † Earth and Environmental Sciences, University of St AndrewsFife, KY16 9AL, UK; ‡ INRA, UMR-ECOFOGKourou, France; § INRA, UMR 1137 Ecologie et Ecophysiologie ForestièresChampenoux, 54280, France; ¶ Research School of Biology, Division of Plant Sciences, Australian National UniversityCanberra, ACT, 0200, Australia

**Keywords:** carbon use efficiency, DALEC, data assimilation, ecosystem respiration, French Guiana, seasonal carbon fluxes, tropical forest

## Abstract

The relative contribution of gross primary production and ecosystem respiration to seasonal changes in the net carbon flux of tropical forests remains poorly quantified by both modelling and field studies. We use data assimilation to combine nine ecological time series from an eastern Amazonian forest, with mass balance constraints from an ecosystem carbon cycle model. The resulting analysis quantifies, with uncertainty estimates, the seasonal changes in the net carbon flux of a tropical rainforest which experiences a pronounced dry season. We show that the carbon accumulation in this forest was four times greater in the dry season than in the wet season and that this was accompanied by a 5% increase in the carbon use efficiency. This seasonal response was caused by a dry season increase in gross primary productivity, in response to radiation and a similar magnitude decrease in heterotrophic respiration, in response to drying soils. The analysis also predicts increased carbon allocation to leaves and wood in the wet season, and greater allocation to fine roots in the dry season. This study demonstrates implementation of seasonal variations in parameters better enables models to simulate observed patterns in data. In particular, we highlight the necessity to simulate the seasonal patterns of heterotrophic respiration to accurately simulate the net carbon flux seasonal tropical forest.

## Introduction

The seasonality of the net carbon flux of Amazonian forests remains uncertain. Existing studies in Amazonian forests have reported both increases (Goulden *et al*., 2004; Hutyra *et al*., 2007; Bonal *et al*., 2008) and decreases (Malhi *et al*., 1998; Chambers *et al*., 2004; Keller *et al*., 2004) in the total carbon sequestered in the dry season. Models struggle to adequately simulate wet-to-dry season changes in the net carbon flux (Saleska *et al*., 2003; Baker *et al*., 2008; Verbeeck *et al*., 2011). The importance of seasonal changes in gross primary production (GPP) and ecosystem respiration (*R*_eco_) on the net carbon flux of tropical forests remains unresolved.

Recent model development studies have focused on improving the simulation of GPP (Fisher *et al*., 2007; Baker *et al*., 2008; Grant *et al*., 2009; Kim *et al*., 2012) rather than the fate of organic matter, and emissions from *R*_eco_. Reco is comprised of autotrophic (leaf, root and stem) and heterotrophic (litter, dead wood and soil) components. Various field studies have estimated the contribution of each component of respiration to total *R*_eco_ (Malhi *et al*., 2009; Metcalfe *et al*., 2010; Malhi *et al*., 2013). However, there is still uncertainty regarding the sensitivity of these individual respiration components to the seasonal drying of soil and how these responses coincide with the seasonality in GPP, to affect seasonal changes in the ecosystem carbon budget (Meir *et al*., 2008).

Carbon use efficiency (CUE) is the proportion of GPP invested into net primary production (NPP), rather than expended as autotrophic respiration (*R*_a_), and is an important indicator of how efficient an ecosystem is at investing assimilated carbon for growth (Waring *et al*., 1998). However, CUE is difficult to quantify accurately using measurements because of uncertainty associated with scaling measurements of leaf, stem and root respiration to the ecosystem scale (Chambers *et al*., 2004). Similarly, estimating CUE remains a challenge for modelling tropical systems because of uncertainties in parameterizing the seasonality of *R*_a_ (Fox *et al*., 2009; Verbeeck *et al*., 2011).

This study reports the responses of a lowland tropical forest to seasonal variations in environmental conditions, at a site in French Guiana, for which multiple ecological time series data sets are available. These time series include: dry and wet season measurements of leaf, stem, soil and coarse woody debris (CWD) respiration; net ecosystem exchange (NEE); litterfall; leaf area index (LAI); woody biomass; and stem growth. The study site experiences a strong seasonal change in soil moisture (Bonal *et al*., 2008; Wagner *et al*., 2011); something which has been predicted to occur over a wider area of Amazonia, particularly the north east, with future climate change (Cox *et al*., 2008; Jupp *et al*., 2010; Marengo *et al*., 2012). The seasonal dry period at our study site has been shown to be coincident with reductions in total *R*_eco_, soil respiration (including root and litter respiration), tree growth, stem respiration and CWD respiration at the site (Bonal *et al*., 2008; Stahl *et al*., 2011; Wagner *et al*., 2012; Rowland *et al*., 2013).

**Figure 1:**
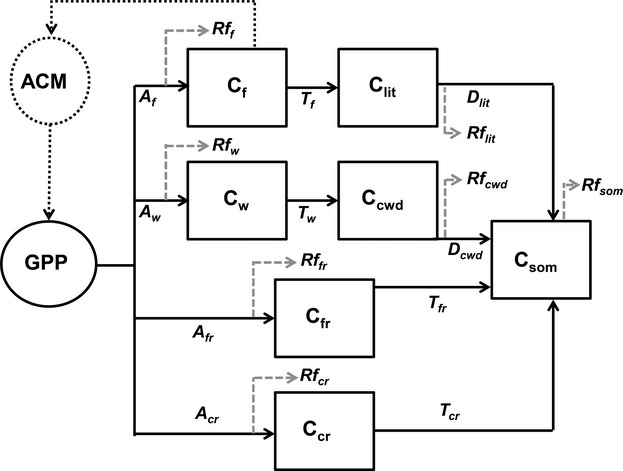
Diagram of the DALEC-FG carbon model, an adaptation of the Data assimilation linked Carbon (DALEC) model (Williams *et al*., 2005). The boxes represent a carbon pool and the arrows represent a carbon flux through the model, the dotted grey arrows represent a loss from respiration, which is set to a fixed fraction of the carbon allocated to each pool. All of the acronyms for the pool and fluxes are explained in the model parameters table (Table 1). The fractions respired from autotrophic pools (foliar carbon; *C*_f_, carbon in wood; *C*_w_, carbon in fine roots; *C*_fr_ and carbon in coarse roots *C*_cr_) are calculated as a fraction of the carbon allocated to the pool. The fraction respired from the litter, coarse woody debris and soil carbon pools (*C*_lit_, *C*_cwd_, *C*_som_) are calculated as a fraction of the total pool.

To achieve the most likely summary of existing data, we adapt the Data Assimilation Linked Carbon Model (DALEC; Fox *et al*., 2009; Williams *et al*., 2005) for use at the site in French Guiana (Fig. 1; hereafter referred to as DALEC-FG). We use Metropolis-Hastings data assimilation (DA; Knorr & Kattge, 2005) to combine uncertain data with the process information and mass balance described by the DALEC-FG model, to constrain the seasonal response of the ecosystem. The DA scheme is used to parameterize the model for both wet and dry season, which are defined using a soil water content threshold (see Methods). Using separate parameters for each season the analysis can attribute, with estimates of uncertainty, the seasonal changes in the net carbon flux to changes in the component carbon fluxes of this tropical forest.

## Materials and methods

### Site

**Figure 2:**
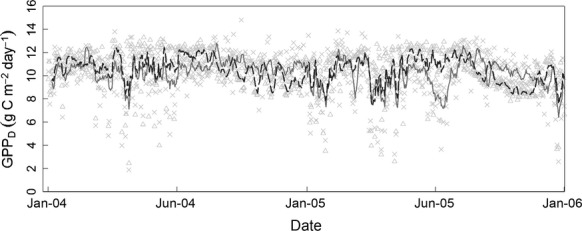
Comparison of the gross primary production (GPP) from the soil–plant–atmosphere model (SPA) run at the Paracou site with the GPP calculated from the eddy covariance data collected at the site from 2004 to 2005 and published in Bonal *et al*. (2008). Light grey crosses indicate daily GPP (g C m^−2^ d^−1^) from Bonal *et al*. (2008) and light grey triangles the equivalent from SPA. The lines show the 6-day running mean from SPA (dark grey dotted line) and Bonal *et al*., 2008 (light grey solid line).

The study focused on a tropical lowland forest site at Paracou Research Station in French Guiana (5°16 N, 52°16 W). Data were collected over a period of 8 years from January 2004 to December 2011 on two adjacent 70 × 70 m *terra firme* permanent forest plots (Bonal *et al*., 2008; Stahl *et al*., 2011, 2013; Wagner *et al*., 2012; Rowland *et al*., 2013). The plots were situated on nutrient-poor acrisols and were similar in ecological characteristics, including species density (103 and 116 species ha^−1^), stem density (612 and 725 stems ha^−1^) and litterfall (7.28 ± 0.3 and 6.42 ± 0.3 Mg ha^−1^ yr^−1^). French Guiana has a strong seasonal rainfall pattern caused by the movement of the intertropical convergence zone twice a year, causing a long (August–November) and short (March) dry season. Consequently, despite the site receiving an average of 3041 mm of rain per year (Gourlet-Fleury *et al*., 2004), during the long dry season rainfall is normally <50 mm per month (Bonal *et al*., 2008). The dry season reduction in rainfall is large enough to causes a significant reduction in leaf water potential (see Supporting Information), and has been shown to have a small effect on GPP (Bonal *et al*., 2008 and see Fig. 2).

### Model description

The DALEC model (Williams *et al*., 2005) was adapted for French Guiana (DALEC-FG) and is a simple box carbon cycle model of carbon pools connected by fluxes (Fig. 1). The original DALEC model has been used in a number of previous modelling studies (Williams *et al*., 2005; Fox *et al*., 2009; Hill *et al*., 2012). Our adaptations to the original DALEC model (Williams *et al*., 2005) included: (i) inclusion of a coarse root pool and a coarse dead wood (CWD) pool (Fig. 1); (ii) Modelling stem, leaf, fine root and coarse root respiration separately (Fig. 1); (iii) Inclusion of a moisture response function to predict heterotrophic respiration created using mean daily soil respiration (*R*_s_) measured at the site (see Supporting Information) and (iv) The use of separate wet and dry season parameters for the allocation, turnover rate and respiration from the foliage, stem and root pools (see below).

As with the original DALEC model, the daily time-step and computational simplicity of DALEC-FG makes it well suited to DA, where a large number of model runs are required. Gross primary productivity (GPP) in DALEC-FG was determined using the Aggregated Canopy Model (ACM; Williams *et al*. (1997); Fig. 1). ACM is an empirical simplification of the Soil–Plant–Atmosphere model (SPA; Fisher *et al*., 2006, 2007; Williams, 1996) which predicts GPP according to daily minimum and maximum temperature, precipitation, radiation, atmospheric CO_2_ concentration, soil water potential, hydraulic resistance, leaf nitrogen and LAI combined with 10 optimized parameters. To ensure ACM was correctly calibrated for the study site, 10 parameters in ACM were optimized to reproduce the GPP predicted by a set of runs performed for the site using the SPA model. SPA, a detailed ecophysiological model, has previously been validated at Amazonian forest sites (Fisher *et al*., 2007). Once SPA was calibrated for our site (see Supporting Information) it accurately produced previously published GPP estimates for this site (Bonal *et al*., 2008; Fig. 2). ACM replicated the SPA GPP with a root mean square error of 0.05 g C m^−2^ d^−1^.

### Soil moisture response function in DALEC-FG

A soil moisture response function for heterotrophic soil respiration was created using *R*_s_ data measured at the site. The *R*_s_ data included respiration from root, litter and soil organic matter. To model the soil water response of heterotrophic respiration, we first had to remove the effect of root respiration from the *R*_s_ data. We estimate root respiration by assuming that it is a constant and that the seasonal changes in soil respiration are caused by heterotrophic processes. Previous studies on our site and at other sites in the eastern Amazon have demonstrated a strong heterotrophic soil respiration response to reductions in soil moisture (Bonal et al., 2008; Metcalfe et al., 2007; Sotta et al., 2007). In comparison, only small, and both positive and negative seasonal changes in autotrophic soil respiration have been found (Metcalfe *et al*., 2007; Da Costa *et al*., 2013). We assume that root respiration is a constant value of 1.9 ± 0.3 g C m^−2^ d^−1^; this is half of the soil respiration when it is averaged over the 2 years of measurements. Root respiration has been shown to be approximately half of annual soil respiration, at our study site (Ponton & Bonal, unpublished data) and at other sites in the eastern Amazon (Metcalfe *et al*., 2007, 2010). To model heterotrophic soil respiration our estimated root respiration value is subtracted from all daily *R*_s_ data (*n* = 601, 2005–2006) and these data are used to create a model of heterotrophic soil respiration.

The seasonal effect of temperature on the heterotrophic respiration from soil was removed by subtracting the change in heterotrophic respiration caused by temperature using the temperature response function in DALEC-FG, which assumes a doubling of respiration rate with a 10 °C rise in temperature. The remaining seasonality in the heterotrophic soil respiration was regressed against the mean measured daily surface soil water content (SWC) which was collected every 30 min in the surface 5–10 cm (see below). A log-normal curve was fitted to these data (Fig. S1) and normalized, so the optimum point (2.5 g C m^−2^ d^−1^) was equal to 1. DALEC-FG was forced with the daily mean of measured SWC data and used this normalized log-normal function to adjust predicted values of carbon loss from the heterotrophic pools based on soil moisture. It should be noted that this moisture response function is an empirical relationship and thus is site specific.

### Defining wet and dry season

Dry season was defined using the soil water content data, including all days where the mean daily SWC was <0.12 m^3^ m^−3^. This threshold was set as the lower quartile of all the SWC data, which had an annual mean and SD of 0.17 ± 0.04 m^3^ m^−3^. In total 733 of 2922 study days were defined as dry season. The wet-dry season division was used to define when the assimilation switched between wet and dry season model parameters for the allocation, turnover time and respiration parameters for the autotrophic carbon pools (foliar carbon (*C*_f_), carbon in wood (*C*_w_) and carbon in fine and coarse roots (*C*_fr_, *C*_cr_)). This seasonal shift meant that the DA could adjust ecosystem dynamics across seasons, testing the hypotheses that seasonal variation in parameters would better enable the model to replicate the observed patterns in the data.

### Data assimilation methodology

The DA scheme optimized 36 parameters. These include separate parameters for the wet and dry season allocation and turnover rate and respiration parameters for the autotrophic pools were included in these 36 parameters (Table 1). A Metropolis-Hastings scheme was used to estimate the *posterior* distribution of model parameters (Knorr & Kattge, 2005). We assume observation errors on different data streams to be uncorrelated and therefore minimize the function: 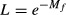


**Table 1:** Parameter descriptions for the DALEC-FG model, including their symbols (s), units, prior value (P), prior lower estimate (PL) and prior upper estimate (PU), the posterior median (Pos), the 15.9th (PosL) and 84.1th (PosU) percentiles on the posterior parameter distributions and sources of the priors estimates for the DALEC-FG model. For allocation, turnover rate and respiration parameters for the autotrophic pools, the wet season posterior parameter values are shown followed by the dry season posterior parameter values in brackets

Parameter	S	Units	P	PL	PU	Pos	PosL	PosU	Source of prior
Initial foliage C stock	*C*_f_	g C m^−2^	384	299	493	421	411	431	Estimated from LMA data & LAI data
Initial wood C stock	*C*_w_	g C m^−2^	23 553	18 343	30 243	22 093	21 015	23 186	See methods section
Initial fine root C stock	*C*_fr_	g C m^−2^	371	289	476	469	373	568	Galbraith 2010
Initial coarse root C stock	*C*_cr_	g C m^−2^	1593	966	2627	2970	1814	4610	Galbraith 2010
Initial litter C stock	*C*_lit_	g C m^−2^	300	182	495	358	264	474	Malhi *et al*. 2009*
Initial coarse wood debris C stock	*C*_cwd_	g C m^−2^	1738	1354	2232	1948	1550	2649	See methods section
Initial soil organic matter C stock	*C*_som_	g C m^−2^	29 000	22 585	37 237	36 820	30 368	45 195	2006
Allocation fraction to foliage	*A*_f_	Fraction of GPP	0.43	0.26	0.71	0.40 (0.31)	0.38 (0.30)	0.42 (0.33)	Malhi *et al*. 2009*
Allocation fraction to wood	*A*_w_	Fraction of GPP	0.26	0.16	0.43	0.24 (0.18)	0.22 (0.17)	0.26 (0.18)	Malhi *et al*. 2009*
Allocation fraction to fine roots	*A*_fr_	Fraction of GPP	0.23	0.14	0.37	0.29 (0.45)	0.25 (0.41)	0.33 (0.47)	Malhi *et al*. 2009*
Allocation fraction to coarse roots	*A*_cr_	Fraction of GPP	0.08	0.05	0.13	0.06 (0.06)	0.04 (0.04)	0.10 (0.09)	Malhi *et al*. 2009*
Turnover rate of foliage†	*T*_f_	Fraction of pool per day	2.4e-3	1.8e-3	3.0e-3	1.7e-3 (2.1e-3)	1.6e-3 (2.0e-3)	1.7e-3 (2.2e-3)	Estimated from LMA and litterfall (see Methods)
Turnover rate of wood†	*T*_w_	Fraction of pool per day	2.5e-5	1.9e-5	3.2e-5	2.2e-5 (2.4e-5)	1.8e-5 (1.9e-5)	2.6e-5 (3.1e-5)	Rutishauser *et al*. 2010
Turnover rate of fine roots†	*T*_fr_	Fraction of pool per day	1.4e-3	6.5e-4	2.9e-3	4.5e-3 (1.5e-3)	3.5e-3 (9.1e-4)	5.7e-3 (2.2e-3)	2006
Turnover rate of coarse roots†	*T*_cr_	Fraction of pool per day	2.5e-5	1.5e-5	4.1e-5	3.8e-5 (2.8e-5)	2.1e-5 (1.8e-5)	6.4e-5 (4.5e-5)	Assumed to be the same as *T*_w_
Turnover rate of litter†	*D*_lit_	Fraction of pool per day	1.0e-3	4.7e-4	2.1e-3	1.1e-3	6.7e-4	2.0e-3	Metcalfe *et al*. 2010
Turnover rate of CWD†	*D*_cwd_	Fraction of pool per day	4.4e-5	2.1e-5	9.3e-5	8.6e-5	4.9e-5	1.3e-4	Carbon lost from CWD per year was calculated using decay rate equations from (Herault *et al*., 2010). Assuming 75% of decayed CWD is respired (Chambers *et al*., 2001) the time to decay whole CWD pool based on 18% of carbon lost to soil pool was then calculated.
Respired fraction of *A*_f_	*R*_Ff_	Fraction of *A*_f_ per day	0.50	0.30	0.82	0.78 (0.96)	0.77 (0.93)	0.79 (0.99)	Default assumption for fraction of respired carbon in ACM
Respired fraction of *A*_w_	*R*_Fw_	Fraction of *A*_fw_ per day	0.50	0.30	0.82	0.61 (0.80)	0.57 (0.77)	0.66 (0.83)	Default assumption for fraction of respired carbon in ACM
Respired fraction of *A*_fr_	*R*_Ffr_	Fraction of *A*_fr_ per day	0.50	0.30	0.82	0.46 (0.33)	0.36 (0.29)	0.53 (0.37)	Default assumption for fraction of respired carbon in ACM
Respired fraction of *A*_cr_	*R*_Fcr_	Fraction of *A*_cr_ per day	0.50	0.30	0.82	0.89 (0.65)	0.61 (0.44)	0.97 (0.84)	Default assumption for fraction of respired carbon in ACM
Respired fraction of *C*_lit_	*R*_Flit_	Fraction of pool per day	1.0e-3	.7e-4	2.1e-3	9.2e-4	4.9e-4	1.7e-3	Set to ACM default
Respired fraction *C*_cwd_	*R*_Fcwd_	Fraction of pool per day	2.0e-4	9.4e-5	4.2e-4	2.3e-4	1.7e-4	2.9e-4	Carbon lost from CWD per year was as for *D*_cwd_. Assuming 75% of decayed CWD is respired (Chambers *et al*., 2001), the fraction of carbon respired from the CWD pool per day was calculated.
Respired fraction *C*_som_	*R*_Fsom_	Fraction of pool per day	1.0e-4	4.7e-5	2.1e-4	6.4e-5	5.2e-5	7.8e-5	Set to ACM default

* Values from Malhi *et al*. (2009) are calculated as averages from the Caxiuanã and Manaus sites only.

† Turnover rate parameters are inserted into the model as a turnover rate (1/(turnover time (yrs)/365).

where L is the likelihood of the model parameters given the data and *M*_f_ is the model data miss fit. *M*_f_ is determined by: 
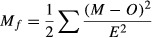
 where *M* is the modelled result, *O* is the observations and *E* is the SE on the observations.

Prior information about the parameter distributions was included using the same form of likelihood function, but comparing parameter selections with estimated prior parameters (Table 1; Knorr & Kattge, 2005). Model parameters were assumed to be real, positive and to have a lognormal probability distribution (Knorr & Kattge, 2005). Therefore, all processes of parameter selection, and acceptance and rejection of parameters in relation to prior ranges were performed in log-normal space (Knorr & Kattge, 2005).

The step size for the DA was set to a random draw from a normal distribution, with a mean of 0 and a SD of 0.004 in log-normal space, resulting in an acceptance rate of 40–45%. The length of the Markov chain was determined using Gelman–Ruben convergence statistic (Brooks & Gelman, 1998). The Gelman–Ruben convergence statistic was calculated using six Markov chains and indicated that after 1 200 000 steps the Markov chain had adequately sampled the posterior distribution, with a convergence level below the 1.2 threshold (Brooks & Gelman, 1998). A burn point – the number of initial accepted parameter combinations which are thrown away – was set at 200 000 to ensure the initial portion of the chain was not sampled. The final posterior distributions for each separate Markov chain was therefore made up of 1 000 000 accepted parameter combinations. The posterior parameter values and ranges were calculated as the 50th, 15.9th and 84.1th percentiles of the 1 million accepted parameter combinations. These percentiles are equivalent to the mean and plus and minus one SD for a log-normal distribution. For data storage purposes the output from 1000 of the 1 million accepted model runs was randomly selected and saved.

### Assimilated data

#### Eddy covariance flux data

Eddy covariance data on a half hourly time-step from 2004 to 2011 were available from a tower located <50 m from our study sites. There is a detailed methodology published for the set-up of the tower (Bonal *et al*., 2008). The NEE data were processed using ALTEDDY software (http://www.climatexchange.nl/projects/alteddy/) and standard quality control checks were used to filter the data (Foken *et al*., 2005). Following all night-time NEE data for which u* values were <0.15 m s^−1^ were filtered out (Bonal *et al*., 2008). As some spurious spikes were still visible in the half hourly carbon flux (FC) and carbon storage data (SFC) all values of SFC and FC greater than 10 SDs were filtered out from the data (in both cases <0.11% of the data were filtered). To create daily values of NEE and limit the use of gap-filled data, only days with ≥40 half hours per day were used. Missing values for these days were replaced with the mean daytime or night-time value for that day, before fluxes were summed. From 2004 to 2011, 497 daily values of NEE were available. Errors for the NEE data were derived from previously published methodologies (Hollinger & Richardson, 2005; Hill *et al*., 2012) (see Supporting information).

#### Foliar data

Leaf respiration measurements were available on our study plots from two studies (Stahl *et al*., 2013; Zaragoza-Castells *et al*., unpublished results). The data included the average and SD of leaf respiration in dark conditions from fully sunlit leaves for 52–70 leaves measured in November 2007, July 2008 and November 2008 (Stahl *et al*., 2013) and from 70 leaves for the dry season of 2010 (Joana Zaragoza-Castells, unpublished data). Leaf respiration data were adjusted to the mean daily temperature over our study period (25.6 °C). These data points were adjusted to a canopy average value by scaling respiration values according to changes leaf respiration between sunlit and shaded leaves (see Supporting Information).

Mean LAI and SD were estimated from measurements made with the Li-2000 (Licor, Lincoln, NE, USA) at between 37 and 49 randomly selected locations per plot in March 2005, November 2005, November 2008, September 2010, March 2011 and September 2011. LAI was compared to model output using an estimate of leaf mass per area (LMA) of 122.07 ± 2.23 g C m^−2^ (where ± indicates SE), measured at the site on 70 leaves (Zaragoza-Castells *et al.,* unpublished results); we assumed half of this mass was carbon.

On our study sites litterfall was measured monthly from January 2004 to December 2011 using four 1 m^2^ litter traps on each plot. Material was collected, dried to a constant mass and then weighed.

#### Woody stem data

Respiration from stems was measured on our study plots (Stahl *et al*., 2011); stem respiration measurements were made over 11 periods, during both wet and dry season, between September 2007 and February 2009. The mean and SE of these measurements were scaled to plot level using surface area of the stems and large branches per unit of ground area (stem area index, SAI; Chambers *et al*., 2004; Robertson *et al*., 2010). The error on stem respiration was derived from the measurement error, following scaling and therefore we assume that the scaling error was captured by the measurement error.

A census of the diameters of all trees ≥10 cm diameter at breast height (DBH, 1.3 m) was conducted in 2004, 2006, 2008 and 2010. These measurements were used to estimate the total aboveground biomass of the plots using a biomass equation for tropical moist forests (Chave *et al*., 2005), which included tree height; tree height was calculated from diameter using a country specific equation (Feldpausch *et al*., 2011). As no error estimation existed for biomass, a SE of 10% of the biomass value was passed into the DA.

Tree diameter growth data were measured 32 times for 114 trees on a monthly to bimonthly basis from 2007 to 2010 on our study plots (Wagner *et al*., 2012). Growth data were not scaled to plot level by Wagner *et al*. (2012) who stated that the trees they measured were not representative of the size structure of the forest. The 11 dry season and 21 wet season growth data measurements from 114 trees from Wagner *et al*. (2012) were used to calculate the ratio of dry to wet season biomass accumulation, which was 0.40 ± 0.09 (where ± indicates SE). These data were assimilated annually to provide the model with information of the approximate magnitude and direction of the seasonal change in woody biomass allocation.

#### Heterotrophic respiration data

Respiration from coarse woody debris (*R*_cwd_) was estimated from 429 measurements made on 33 samples during 13 periods from July 2011 to November 2011(Rowland *et al*., 2013). Full details of measurements and method used to scale the *R*_cwd_ measurements to a plot level are available in Rowland *et al*.(2013).

Automatic soil respiration (*R*_s_) data at the study site were measured from April 2005 to December 2006 (Bonal *et al*., 2008 and Ponton & Bonal, unpublished data). *R*_s_ was measured every half hour on the study site using four automated chambers (Bonal *et al*., 2008). The chambers were placed on top of the surface litter and respiration measurements therefore represent the combined respiration from surface litter, root litter and root and soil. Half hourly values were then averaged into daily values. Error was derived from the SE on the four-chamber measurements. Data were only used when three or more of the soil chambers recorded measurements (577 days). There was significant autocorrelation in the *R*_s_ data, this was removed by filtering the data to every 30 days (Gomez Dans, 2004) (*n* = 19). To maintain consistency with the assumptions made in the modelled soil moisture response, we assimilate *R*_s_ data which has been separated into autotrophic and heterotrophic components, described earlier in the methods.

#### Soil water content data

Soil water content data were taken every 30 min from two probes at the study sites. For 2004–2008, data were available from a frequency domain sensor (CS615; Campbell Scientific Inc., North Logan, UT, USA) at 0.05 m depth 15 m from the flux tower. Data were available from a second frequency domain sensor (CS616; Campbell Scientific Inc.) inserted at 0.10 m depth, 10 m from the flux tower for 2007–2011. These data sets were averaged into daily values and corrected for the effects of different probe depth (see Supporting Information).

#### Steady-state observations, error estimation and model output

The model in its standard form makes no assumption of steady state. These primary forests are likely to be relatively close to steady state over decadal timescales. Therefore, to ensure that the modelled carbon pools were close to steady state, we assimilated seven additional pseudo-observations which were the change in size of each of the seven carbon pools in the DALEC-FG model. These observations had a value of 0 and a SD of 2% of the size of the pool. This solution was necessary because computational limits prevented running the model until it was in steady state, as part of the assimilation process.

SE was used as an estimate of uncertainty on the assimilated data (Richardson *et al*., 2010). When combining errors (e.g. multiplying leaf respiration by LAI), the errors were assumed to be random and uncorrelated (Hughes & Hase, 2010). The number of data points for each assimilated data stream and the average error for each data stream are shown in Table 2.

**Table 2:** The number of data points contributing to each data stream used in the DA and the average error on these data (SE, gC m^−2^ d^−1^)

Data stream	No.	SE
Net ecosystem exchange	497	2.66
Leaf respiration	4	0.76
Leaf area index	6	0.44
Litterfall	112	0.20
Stem respiration	11	0.08
Aboveground biomass	4	2258.35
Soil respiration	19	0.52
Coarse dead wood respiration	13	0.07

### Prior information

Where possible priors on states and parameters were based on data from published sources and unpublished data from the study site. Where site data were not available, estimates from nearby sites in northern Brazil were used. Where no data existed the parameters were set to a best approximation or to the default values from the DALEC model (Williams *et al*., 2005). All the prior values were assigned a SD of 0.25, 0.5 or 0.75 in log-normal space (Knorr & Kattge, 2005); Table 1). SD values were assigned based on an assessment of the uncertainty of the data source and on creating realistic limits on the mean estimate.

## Results

**Figure 3:**
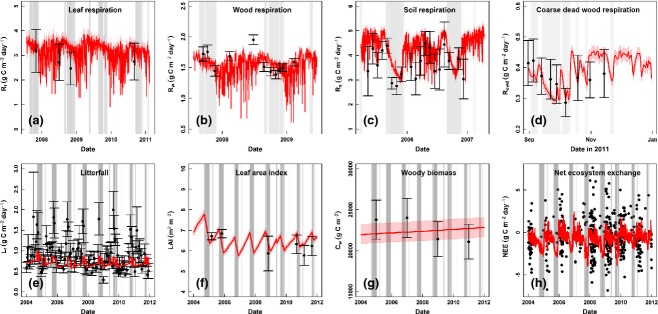
Comparison of data (black points, shown with standard error bars) with model output from the DA. Median results (red line) with the 15.9th and the 84.1th percentiles (red shaded area), which represent 1 SD for nongaussian distributions, are shown for the results of the DA. The grey shaded area indicates the periods classified as the dry season.

The results of the analysis show that mean annual GPP is 3756.7 ± 19.1 gC m^−2^ yr^−1^, 9.1% greater than *R*_eco_ (3415.3 ± 38.5 gC m^−2^ yr^−1^); demonstrating that this forest stores carbon on an annual basis. However, our analysis demonstrates that the strength of the carbon sink increases by approximately four times from wet (NEE: −0.54 ± 0.12 gC m^−2^ d^−1^) to dry season (NEE: −2.1 ± 0.15 gC m^−2^ d^−1^; Table 3; Fig. 3). The increased strength of the sink was caused by a 0.79 ±0.07 gC m^−2^ d^−1^ increase in GPP in response to higher dry season radiation and a simultaneous decrease of 0.78 ± 0.20 gC m^−2^ d^−1^ in *R*_eco_. The effects of decreasing respiration and increasing GPP were therefore equally important for the seasonal change in the net carbon flux of this ecosystem. The seasonal reduction in *R*_eco_ was caused by a reduction in heterotrophic respiration (*R*_h_), which not only caused the decrease in *R*_eco_ but also compensated for an increase in autotrophic respiration of 0.30 ± 0.22 gC m^−2^ d^−1^ (*R*_a_; Table 3).

**Table 3:** The mean carbon pools and fluxes predicted by the DA analysis for study site from 2004 to 2011. Data are shown as mean values for wet and dry season and as mean annual sums. The values are calculated from 1000 randomly selected DA model runs and shown alongside the SD across these model runs (SD)

	Wet season		Dry season		Annual	
	Mean	SD	Mean	SD	Sum	SD
Allocation	gC m^−2^ d^−1^				gC m^−2^ yr^−1^	
*A*_f_	4.01	0.19	3.42	0.18	1413.1	54.9
*A*_w_	2.36	0.12	1.88	0.07	818.5	38.5
*A*_fr_	3.04	0.22	4.84	0.22	1272.6	61.7
*A*_cr_	0.64	0.14	0.71	0.18	252.5	43.7
Respiration	gC m^−2^ d^−1^				gC m^−2^ yr^−1^	
*R*_f_	3.13	0.18	3.27	0.15	1158.9	54.2
*R*_w_	1.48	0.03	1.53	0.03	544.2	8.5
*R*_fr_	1.42	0.17	1.40	0.15	501.3	53.7
*R*_cr_	0.49	0.16	0.64	0.14	210.8	54.9
*R*_lit_	0.40	0.09	0.26	0.06	130.8	30.2
*R*_cwd_	0.41	0.02	0.26	0.01	134.5	6.5
*R*_som_	2.23	0.16	1.43	0.11	735.0	54.6
Ecosystem fluxes	gC m^−2^ d^−1^				gC m^−2^ yr^−1^	
NEE	−0.54	0.12	−2.11	0.15	−341.4	36.3
GPP	10.09	0.05	10.87	0.05	3756.7	±±
*R*_eco_	9.55	0.13	8.77	0.15	3415.3	38.5
*R*_a_	6.53	0.17	6.83	0.14	2415.1	49.7
*R*_h_	3.02	0.12	1.93	0.08	1000.2	39.1
CUE	0.35	0.02	0.37	0.01	0.36	0.02
Stocks	gC m^−2^				gC m^−2^	
*C*_f_	398	8	397	8	398	8
*C*_w_	22376	1225	22362	1217	22373	1223
*C*_fr_	465	57	520	52	480	56
*C*_cr_	2842	717	2841	714	2842	717
*C*_lit_	524	63	530	63	525	64
*C*_cwd_	2181	364	2179	364	2181	364
*C*_som_	29579	5668	29462	5676	29550	5670

**Figure 4:**
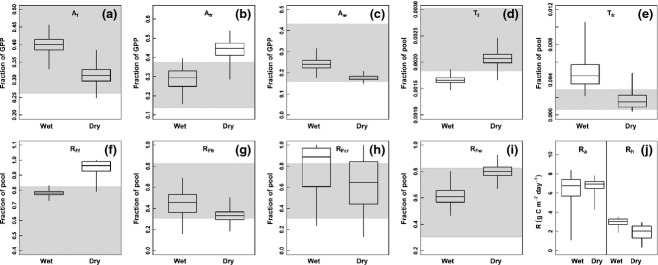
Box plots of the DA posterior parameter estimates for the allocation (a–c), turnover (d–e) and respiration (f–i) parameters which showed dry and wet season differences. The grey shaded area shows the prior ranges for the parameter values (see Table 1). Panel J shows the effect of these parameter changes on the modelled autotrophic respiration (*R*_a_, g C m^−2^ d^−1^) in the wet and dry season (left), relative to the seasonal change in the heterotrophic respiration (*R*_h_, g C m^−2^ d^−1^; right).

The analysis tightly constrained (SDs <10% of the mean) the GPP, *R*_eco_, *R*_a_, *R*_h_ and CUE fluxes (Table 3). Mean annual *R*_a_ from the analysis was 2415 ± 50 gC m^−2^ yr^−1^, more than twice the size of the annual *R*_h_ (1000 ± 39 gC m^−2^ yr^−1^; Table 3). The *R*_h_ : *R*_a_ ratio decreased from 0.46 ± 0.02 in the wet season to 0.28 ± 0.01 (Table 3, Fig. 4). This seasonal change was caused by the 36% reduction in dry season *R*_h_. Total *R*_a_ only increased by 4% from wet to dry season; however, the reduction in dry season *R*_h_ resulted in *R*_a_ comprising 80% of the dry season *R*_eco_. Mean annual carbon use efficiency (CUE) was 0.36 ± 0.02, but increases from wet to dry season by 5.38 ± 0.3%.

On an annual basis similar proportions of GPP were allocated to foliage (37.7 ± 1.5%) and fine roots (33.9 ± 1.7%; Table 3). The remainder of GPP was allocated to stem wood (21.8 ± 1.0%) and coarse roots (6.7 ± 1.2%). However, the division of carbon allocation among leaves, coarse wood (which includes both stems and coarse roots) and fine roots varied significantly when analysed at a seasonal timescale. The results of the DA indicate increased allocation of carbon to coarse wood and foliage in the wet season, and greater allocation to fine roots in the dry season (Fig. 3; Tables 1 and 2). These changes were driven by significant changes to the allocation parameters from the wet to dry season; *A*_f_ and *A*_w_ decreased 22.5 ± 3.1% and 25 ± 4.4%, respectively, from wet to dry season, whereas *A*_fr_ increased 35.5 ± 10% (Fig. 4, Table 1).

There were distinct seasonal differences in nine of the 12 parameters associated with the autotrophic pools (Fig. 4). Increases in the respired fraction of the foliar and wood pools from wet to dry season (18.75 ± 1.3%, and 23.75 ± 3.9% respectively) were contrasted by decreases in the fraction respired from the fine and coarse root pools (28.3 ± 12.5% and 27.0 ± 19.9% respectively). The analysis predicted high uncertainty (SD ≥ 40% of the mean) for certain parameters: the allocation of carbon to coarse roots, and the turnover of coarse and fine roots, and coarse dead wood and litter (Fig. 4 and Table 1). The errors on the posterior parameter distributions and the simulated model output associated with both the fine and coarse root pools were consistently greater than those associated with the foliage and stem pools (Table 1 and 2; Fig. 4). However, despite a significant increase in the turnover rate of foliage and therefore litterfall in the dry season (Fig. 4d), the DA still remained unable to simulate the high litterfall values which occurred at this site during a 1–2 month period in early to middry season (Fig. 3e). The litterfall data therefore remained the most poorly fitted data in this study (Fig. 3e).

## Discussion

This is the first study which uses DA to optimize separate wet and dry season parameters in a tropical forest and to investigate how fluxes from different forest components contribute to seasonal changes in net ecosystem carbon flux. The implementation of seasonal variations in parameters provides a mechanism through which the DALEC-FG carbon model is able to better simulate the observed patterns in flux data. The analysis determines that four times more carbon is sequestered in the wet than the dry season in the seasonal tropical forest studied, and that there are significant seasonal changes in carbon allocation, and CUE.

The fourfold increase in the net carbon sequestration (391.1 ± 91.2% decrease in NEE; Table 3) in dry season was the result of the response of heterotrophic respiration to soil moisture and an increase in GPP in response to increased solar radiation. The increase in NEE in the dry season is larger than has been modelled for other tropical humid forest sites in northern Brazil (Baker *et al*., 2013). Our estimated values of annual *R*_a_ and *R*_h_ were similar to estimates from empirical bottom-up net carbon flux studies elsewhere in eastern Amazonian forests (Malhi *et al*., 2009; Metcalfe *et al*., 2010). The reduction in *R*_h_ from wet to dry was driven by a modelled response to reduced soil water availability (see Methods). Without this modelled moisture response, *R*_h_ increased in the dry season in response to increased dry season temperature (data not shown) and consequently the seasonality of the soil respiration was incorrectly simulated, resulting in an underestimation of dry season carbon sequestration and an inability to match the seasonality of NEE.

The low wet to dry season variation in average GPP (Table 3) and the stronger variation in *R*_eco_ matched patterns observed by Bonal *et al*. (2008) at this site. In 2004, our GPP estimate was 2.74% greater and in 2005, 5.74% greater than previously estimated from eddy covariance data at the site (Bonal *et al*., 2008). In contrast, our *R*_eco_ estimates were 3.37% lower in 2004 and 1.41% lower in 2005 than estimates from Bonal *et al*. (2008). Considering the errors associated eddy covariance measurements (Bonal *et al*., 2008; Hutyra *et al*., 2008) these differences are low. However, such differences result in our estimates of carbon sequestered by this ecosystem being 2.18 times greater in 2004 and 1.58 times greater in 2005 than previously estimated by eddy covariance data (Bonal *et al*., 2008). However, in this study, we are able to determine with an assessment of uncertainty, the importance of the seasonality of *R*_h_, GPP and components of *R*_a_ for altering carbon sequestration and CUE estimates of tropical forests.

Carbon use efficiency (0.36 ± 0.02) was lower than temperate forest values of ca. 0.5 (Waring *et al*., 1998) and closer to the CUE values proposed for two undisturbed old-growth forests in the eastern Amazon (0.34 ± 0.10 and 0.34 ± 0.07; Malhi *et al*., 2009). The 5% increase in CUE in the dry season was caused by a greater dry season increases in GPP (8%) than in *R*_a_ (4%; Table 3) suggesting that this forest is more efficient at investing carbon in the dry season, when GPP is elevated because of higher solar incident radiation.

The relatively even annual distribution of GPP between foliage, fine root and coarse wood (stems and coarse roots) is consistent with a synthesis of 35 old-growth rain forests across the Amazon (Malhi *et al*., 2011). However, the DA demonstrates that there is a wet to dry season shift from greater allocation into stems and foliage, to greater allocation into fine roots (Fig. 4, Table 3). Such a seasonal change in allocation is consistent with a general adaptive strategy to overcome soil drought (Nepstad *et al*., 1994; Brando *et al*., 2008).

Root respiration and turnover showed high uncertainty in this study (Fig. 4, Table 3). In general, we found that parameters associated with both coarse and fine root had consistently greater error than those associated with the woody of foliage pools (Fig. 4, Table 1). Such uncertainty resulted from a lack of data to explicitly constrain the allocation and turnover of these pools, in combination with high errors on the prior estimates for these parameters from the literature (Table 1). More field data are therefore necessary to provide a tighter constraint on the seasonal changes in patterns of root dynamics; available methodologies to follow these patterns are destructive and involved heavy investments and have been seldom applied in tropical forests so far.

The model used in this study is a simple approximation of the complex processes which determine seasonal changes in the carbon balance of a tropical forest. The simple model representation required for the DA leads to structural limitations in the DALEC-FG model; for example, a threshold change in model parameterization between wet and dry season does not reflect, what is likely to be a gradual shift in ecosystem function. Also, the absence of certain ecological processes may have affected the results, for example, the absence of nonstructural carbohydrates, root exudates in DALEC-FG may have altered the seasonal changes in GPP and R_a_. Similarly, we acknowledge that small amounts of variation in our assumptions that root respiration is constant and comprises half of total soil respiration, may have substantial effects on our results and further research is necessary to test such assumptions. However, with the available data and information from the literature (see Methods) our model of soil respiration provided the best possible estimation of the response of soil respiration at this site. Unfortunately, model simplification is necessary for DA, however, it can be used to highlight key areas of model function which requires future development.

The simple division of leaf turnover into a dry season and a wet season rate was insufficient to capture the large pulse of litterfall that is observed during the first 1 or 2 months of the dry season (Fig. 3e). The model could not simulate seasonal litterfall without causing a seasonal pattern in LAI, which was not observed in the LAI data available at this site (Fig. 3f). However, it is possible that there was a short-term change in the LAI following the litterfall pulse and therefore higher resolution LAI data are necessary. Recent studies have developed improved litterfall models at three sites across the Amazon, which were able to reproduce a more realistic pulse of litterfall in the dry season (De Weirdt *et al*., 2012; Kim *et al*., 2012), as observed across multiple sites in the tropics (Chave *et al*., 2010). However, phenology still remains difficult to model in the tropics (Verbeeck *et al*., 2011; De Weirdt *et al*., 2012; Kim *et al*., 2012) and it is important to consider that simplified leaf-fall models such as the turnover of leaves in DALEC-FG are insufficient for tropical regions. The simple leaf-fall model may have bias some of our results; for example, an underestimation of litterfall could lead to an underestimation of heterotrophic respiration from litter.

Few DA studies have focused on tropical forests and no other study has used such a comprehensive set of time-series data to constrain the seasonality of the carbon budget of a tropical forest system. This study demonstrates that the implementation of seasonal variations in parameters can provide a mechanism through which models can better simulate observed patterns in carbon fluxes at tropical forest sites; however, we caution that replicating DA at other sites across the Amazon is necessary to test this more broadly. We show that it is necessary to simulate the response of heterotrophic respiration to soil moisture to accurately model both the annual and seasonal changes in the net carbon flux of forests which experience strong seasonal changes in precipitation and radiation. The DA analysis tightly constrained the GPP, NEE, *R*_eco_, *R*_a_, *R*_h_ and CUE at a tropical forest site in the north east Amazon. Consequently, we demonstrate that this forest sequesters four times as much carbon in the dry season as in the wet season as a result of an increase in GPP and a decrease in *R*_h_, which more than compensates for a small dry season increase in *R*_a_. Consistent with a general strategy to avoid drought stress, the DA also indicated a shift from greater allocation to foliage and wood in the wet season and greater allocation to fine roots in the dry season. This study uses a novel technique, which has shown that using multiple data streams to optimize separate dry and wet season model parameters can significantly improve a model’s ability to predict the effects of seasonal drought on tropical forest carbon fluxes.

## Abbreviations

*A*_F*n*_: GPP fraction allocated to pool *n*
*A*_*n*_: Allocation of carbon to pool *n*
*C*_*n*_: Carbon stock for pool *n*
*Cr*: Coarse roots
CUE: Carbon Use Efficiency
*CWD*: Coarse woody debris
*f*: Foliage
*fr*: Fine roots
GPP: Gross Primary Production
LAI: Leaf Area Index
*L*_*f*_: Litterfall
*Lit*: Litter
*R*_a_: Autotrophic respiration
*R*_eco_: Ecosystem Respiration
*R*_F*n*_: Respired fraction of carbon pool *n*
*R*_h_: Heterotrophic respiration
*R*_*n*_: Respiration from carbon pool *n*
*SOM*: Soil organic matter
*T*_*n*_: Turnover rate of carbon from pool *n*
*w*: Wood

## Supporting Information

Figure S1 Relationship between daily average soil water content (SWC m^3^ m^−3^) from surface 10 cm and heterotrophic soil respiration (*R*_s__hetero_). *R*_s__hetero_ is derived from the measured daily average soil respiration (g C m^−2^ d^−1^) corrected to remove the effects of temperature response and respiration from roots, shown in grey points. A log-normal curve is fitted through these points (black line; y= c*(1/((swc+d)*sqrt(2pi.a^2^)))*exp -((log(swc+d)-b)^2^/(2 a^2^))), where a=1.04, b=-1.45, c=0.088 and d=-0.08.

Data S1 Supporting information for the methods section.
