# Supplementary figures and images for: Evidence for strong seasonality in the carbon storage and carbon use efficiency of an Amazonian forest
Source: Glob Chang Biol. 2014 Jan 20;20(3):979–91. doi: 10.1111/gcb.12375 (PMC4298765; doi:10.1111/gcb.12375)

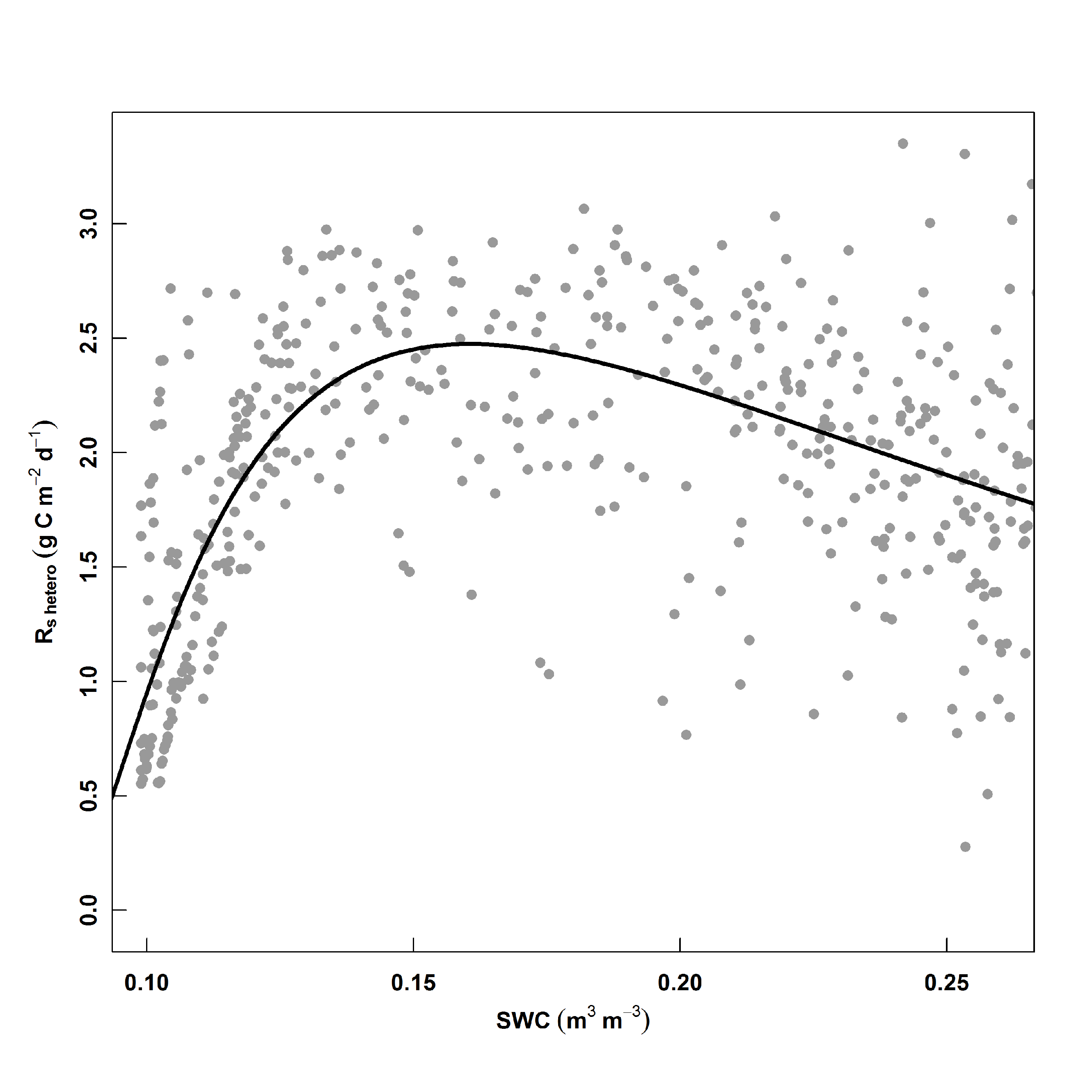

Supplement: Figure S1 — Relationship between daily average soil water content (SWC m3 m−3) from surface 10 cm and heterotrophic soil respiration (Rs hetero). Rs hetero is derived from the measured daily average soil respiration (g C m−2 d−1) corrected to remove the effects of temperature response and respiration from roots, shown in grey points. A log-normal curve is fitted through these points (black line; y= c*(1/((swc+d)*sqrt(2pi.a2)))*exp -((log(swc+d)-b)2/(2 a2))), where a=1.04, b=-1.45, c=0.088 and d=-0.08. [file gcb0020-0979-sd1.tiff]
