# Supplementary material for: Evidence for strong seasonality in the carbon storage and carbon use efficiency of an Amazonian forest
Source: Glob Chang Biol. 2014 Jan 20;20(3):979–91. doi: 10.1111/gcb.12375 (PMC4298765; doi:10.1111/gcb.12375)
Supplement: Data S1 — Supporting information for the methods section. [file gcb0020-0979-sd2.docx]

**Supporting Information**

**Leaf water potential**

Stahl et al., (2012) measured leaf water potential (LWP) on 9 trees of 7 species during the wet season of 2007 and 2008, and during dry season 2008. From these trees the average LWP was: -0.41±0.03 MPa in the dry season of 2007, -0.59±0.06 MPa in the dry season of 2008 and -0.23±0.02 MPa in the wet season of 2008. Consequently the LWP was significantly greater in the wet season of 2008 than the dry season of 2007 (P<0.01, Wilcoxon rank test) and the dry season of 2008 (P<0.01, Wilcoxon rank test).

**Calibration of the SPA model**

SPA was run on a half hourly time-step for our study area over the period 2004-2011. SPA was calibrated using detailed leaf data recorded at the site in 2011 (Bloomfield, 2012, Zaragoza-Castells *et al.*, in prep) which suggested the maximum rate of carboxylation (*V*_cmax_) was 52.4±1.9 µmol m^-2^ s^-1^, and the maximum rate of electron transport (*J*_max_) was 63.7±2.2 µmol m^-2^ s^-1^, leaf mass per area (LMA) was 122.1±2.2 g m^-2^, and leaf nitrogen content was 1.9±0.1 g m^-2^. In addition minimum leaf water potential to -2.5 MPa (Fisher *et al.*, 2006). The clay and sand contents used to calibrate the soils in SPA were taken from data collected at the site (Epron *et al.*, 2006). Other parameters for the SPA model are reported in studies which calibrated the model against detailed physiological measurements from a tropical forest site in the eastern Amazon (Fisher *et al.*, 2007, Fisher *et al.*, 2006). Once SPA was calibrated a series of runs with a range of LAIs from 3–10 m^2^ m^-2^ were performed, to ensure that the optimisation of the aggregated canopy model (ACM; Williams *et al.,* 1997) was not specific to a specific LAI.

**Creating errors for NEE data**

Estimates of error on NEE data were necessary to determine the confidence in the data estimation. The DA uses data errors when determining how close model simulations are to the data. NEE error estimates were generated on a half hourly time-step (Hollinger & Richardson, 2005). The half hourly error estimates were divided into 12 categories based on month of the year to account for seasonal changes in the error. When plotted, the errors for each month formed a double Laplacian distribution, from which 48 points were randomly sampled and added to produce a daily error estimate (Hill *et al.*, 2012). This sampling was repeated 50,000 times, and on error estimated from each month and the standard deviation of the samples was used as the estimation of the monthly error on the NEE values (Hill *et al.*, 2012).

**Scaling leaf respiration data**

We estimated sunlit and shaded leaf fractions of the canopy using the following equation (Doughty & Goulden, 2008, Malhi *et al.*, 2013):


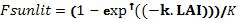
$Fsunlit=(1-e^{-k\cdot LAI})/k$ *Equation S1*

K is the light extinction coefficient and Fsunlit is the sunlit leaf fraction. k is 0.5/cos(Z); where Z is the solar zenith angle. We assume the solar zenith angle to be 30° (Malhi *et al.*, 2013). Equation S1 assumes a spherical leaf angle distribution, as is assumed by the SPA and ACM models. Using this equation with our LAI measurements for 2008, the equation estimates that 1.69 m^2^ m^-2^ of the LAI was sunlit and 4.6 m^2^ m^-2^ was shaded. To account for lower respiration in lower canopy leaves estimates from the Tapajós forest, Brazil (Domingues *et al.*, 2007), were used, which showed that respiration in the mid canopy was ~43% of the values of the fully sunlit upper canopy. The inhibition of leaf respiration during the daytime (Atkin *et al.*, 2000, Atkin & Macherel, 2009) was accounted for by assuming that leaf respiration during daytime hours (12 hours per day) was inhibited by 33%. This figure was obtained by applying a reported equation for light inhibition of leaf respiration (Atkin *et al.*, 2000) to a plot in the Tapajós forest in Brazil (Lloyd *et al.*, 2010, Malhi *et al.*, 2009). This method has been previously applied in other papers modelling carbon exchange in tropical forests (Malhi *et al.*, 2009, Malhi *et al.*, 2013). The error on the leaf respiration data was calculated through propagating errors from leaf level respiration, and LAI measurements(Hughes & Hase, 2010).

**Correcting soil water content data for depth**

To get continuous soil water content data series data for 2007-2011 from the CS616 probe were averaged into daily mean values and combined with mean daily SWC values from the CS615 probe for 2004-2006. The data from the CS615 probe were corrected for the effects of differences in probes and depth. Daily mean SWC values from the CS615 were depth adjusted using an empirical regression between the mean daily values of SWC from the two probes during 2007 (r^2^=0.82, p<0.001). Three were 72 gaps in the daily 2004-2011 SWC dataset; mean gap length was 8±9 days. These gaps were filled, where possible, with corrected data from the CS615 probe (n=52), otherwise the gaps were filled using the average of the 3 data points either side of the gap (n=20).

References

Atkin OK, Evans JR, Ball MC, Lambers H, Pons TL (2000) Leaf respiration of snow gum in the light and dark. interactions between temperature and irradiance. *Plant Physiology,* **122**, 915-923.

Atkin OK, Macherel D (2009) The crucial role of plant mitochondria in orchestrating drought tolerance. *Annals of Botany,* **103**, 581-597.

Bloomfield KJ (2012) The nature of photosynthetic phosphorus limitations for tropical tree species Unpublished PhD The University of Leeds.

Domingues TF, Martinelli LA, Ehleringer JR (2007) Ecophysiological traits of plant functional groups in forest and pasture ecosystems from eastern Amazônia, Brazil. *Plant Ecology,* **193**, 101-112.

Doughty CE, Goulden ML (2008) Seasonal patterns of tropical forest leaf area index and CO(2) exchange. *Journal of Geophysical Research-Biogeosciences,* **113**.

Epron D, Bosc A, Bonal D, Freycon V (2006) Spatial variation of soil respiration across a topographic gradient in a tropical rain forest in French Guiana. *Journal of Tropical Ecology,* **22**, 565.

Fisher RA, Williams M, Da Costa AL, Malhi Y, Da Costa RF, Almeida S, Meir P (2007) The response of an Eastern Amazonian rain forest to drought stress: results and modelling analyses from a throughfall exclusion experiment. *Global Change Biology,* **13**, 2361-2378.

Fisher RA, Williams M, Do Vale RL, Da Costa AL, Meir P (2006) Evidence from Amazonian forests is consistent with isohydric control of leaf water potential. *Plant, Cell & Environment,* **29**, 151-165.

Hill TC, Ryan E, Williams M (2012) The use of CO2 flux time series for parameter and carbon stock estimation in carbon cycle research. *Global Change Biology,* **18**, 179-193.

Hollinger DY, Richardson AD (2005) Uncertainty in eddy covariance measurements and its application to physiological models. *Tree Physiology,* **25**, 873-885.

Hughes IG, Hase TPA (2010) *Measurements and their Uncertainties A Practical Guide to Modern Error Analysis. ,* Oxford, UK, Oxford University Press.

Lloyd J, Patino S, Paiva RQ *et al.* (2010) Optimisation of photosynthetic carbon gain and within-canopy gradients of associated foliar traits for Amazon forest trees. *Biogeosciences,* **7**, 1833-1859.

Malhi Y, Aragao LEOC, Metcalfe DB *et al.* (2009) Comprehensive assessment of carbon productivity, allocation and storage in three Amazonian forests. *Global Change Biology,* **15**, 1255-1274.

Malhi Y, Farfán Amézquita F, Doughty CE *et al.* (2013) The productivity, metabolism and carbon cycle of two lowland tropical forest plots in SW Amazonia, Peru. *Plant ecology and Diversity*.

Williams M, Rastetter EB, Fernandes DN, Goulden ML, Shaver GR, Johnson LC (1997) Predicting gross primary productivity in terrestrial ecosystems. *Ecological Applications,* **7**, 882-894.

Zaragoza-Castells J, Meir P., Atkin O. *et al.* (in prep) How does nutrient availabity influence rates of leaf respiration across Tropical Rainforest?
